# Cost-effectiveness analysis of toripalimab plus bevacizumab as first-line therapy for advanced hepatocellular carcinoma

**DOI:** 10.3389/fpubh.2026.1649775

**Published:** 2026-03-30

**Authors:** Chuangao Wu, Tingfang Wei, Xingfa Ye, Zhuqing Dong, Xiulan Chen

**Affiliations:** Mindong Hospital Affiliated to Fujian Medical University, Ningde, Fujian, China

**Keywords:** bevacizumab, cost-effectiveness, first-line treatment, hepatocellular carcinoma, toripalimab

## Abstract

**Background:**

The HEPATORCH trial demonstrated that toripalimab plus bevacizumab (TOR-BEV) offers superior survival outcomes for patients with advanced hepatocellular carcinoma (HCC) compared to sorafenib. However, the significantly higher costs of toripalimab and bevacizumab raise concerns about the cost-effectiveness of TOR-BEV. This study evaluates the cost-effectiveness of TOR-BEV as a first-line treatment for advanced HCC within the context of China’s healthcare system, relative to sorafenib.

**Methods:**

A partitioned survival model with three health states was developed to compare the cost-effectiveness of TOR-BEV and sorafenib as first-line therapies for advanced HCC. Clinical data were sourced from the HEPATORCH trial. Drug costs were based on national tender prices, while other costs and utility parameters were obtained from the literature. The primary outcomes were total costs, quality-adjusted life-years (QALYs), and incremental cost-effectiveness ratios (ICERs). Sensitivity analyses were conducted to assess the robustness of the results.

**Results:**

The TOR-BEV regimen generated 1.88 QALYs at a cost of $42,239.26, whereas sorafenib resulted in 1.43 QALYs at a cost of $11,201.54. The ICER for TOR-BEV compared to sorafenib was $69,231.90 per QALY gained, exceeding the predefined willingness-to-pay threshold of $40,365 per QALY. The probability of TOR-BEV being deemed cost-effective was only 1.5%. Key factors influencing the model outcomes included the utility value of progression-free survival, weight, the cost of bevacizumab, the utility value of progressive disease, and the cost of toripalimab.

**Conclusion:**

From the perspective of the Chinese healthcare system, TOR-BEV is unlikely to be cost-effective as a first-line treatment for advanced HCC compared to sorafenib unless the prices of both toripalimab and bevacizumab are reduced to below 50.1% of their current prices. However, this regimen was cost-effective in the subgroup of patients with an Eastern Cooperative Oncology Group performance status score of 1, and economic viability is also expected in highly developed regions such as Beijing.

## 1 Introduction

Globally, primary liver cancer is the sixth most prevalent malignancy and the third leading cause of cancer-related deaths (1). Hepatocellular carcinoma (HCC), which accounts for approximately 90% of primary liver cancers, is characterized by high aggressiveness, rapid progression, and a poor prognosis (2). China reports a disproportionately high incidence of HCC, contributing to nearly half of the world’s new cases each year (3). The overall prognosis for patients with HCC remains poor, with a 5-year survival rate of only 19.6% (4). Additionally, the majority of HCC cases are diagnosed at advanced stages, with these patients having a 5-year survival rate of under 10% (5, 6). Over the last decade, targeted therapies based on lenvatinib and sorafenib have become the standard first-line treatments for advanced HCC. Although these therapies have improved patient outcomes, they have only extended the median overall survival (OS) to 10–15 months (7, 8). This highlights the urgent need for more effective treatment strategies for advanced HCC.

Numerous clinical trials have demonstrated that immune checkpoint inhibitors (ICIs), in combination with anti-angiogenic agents such as bevacizumab, outperform sorafenib in prolonging progression-free survival (PFS) and OS in patients with advanced HCC (9–11). The recently completed phase III HEPATORCH trial investigated the efficacy and safety of toripalimab (an ICI) plus bevacizumab (TOR-BEV) as a first-line treatment for advanced HCC. The trial found that TOR-BEV significantly improved the median PFS to 5.8 months, compared to 4.0 months with sorafenib, and increased the median OS to 20.0 months, compared to 14.5 months with sorafenib. Both treatment regimens exhibited comparable safety profiles (12). Thus, TOR-BEV represents a promising new option for the treatment of advanced HCC.

Since 2018, the National Healthcare Security Administration of China has formally incorporated pharmacoeconomic evaluation into the negotiation process for dynamic adjustments to the National Reimbursement Drug List, establishing it as a critical tool for determining whether high-value innovative medicines can be included in the list. This mechanism ensures clinical accessibility while achieving a balance between healthcare fund sustainability and patient benefits. Given the constraints on medical resources in China (13), policymakers and clinicians must carefully consider the cost-effectiveness of treatment options to ensure optimal resource allocation. This is particularly critical in cancer care, which is associated with high treatment costs (14). While TOR-BEV offers superior survival benefits compared to sorafenib, it also incurs significantly higher treatment costs. Therefore, evaluating whether TOR-BEV is cost-effective as a first-line treatment for advanced HCC is essential. To date, no economic evaluations of TOR-BEV as a first-line treatment for advanced HCC have been conducted. This study aims to fill this gap by assessing the cost-effectiveness of TOR-BEV, in comparison to sorafenib, from the perspective of China’s healthcare system.

## 2 Methods

This study was conducted following the updated Consolidated Health Economic Evaluation Reporting Standards guidelines (2022 version, Supplementary Table 1) (15).

### 2.1 Model construction

**Figure 1:**
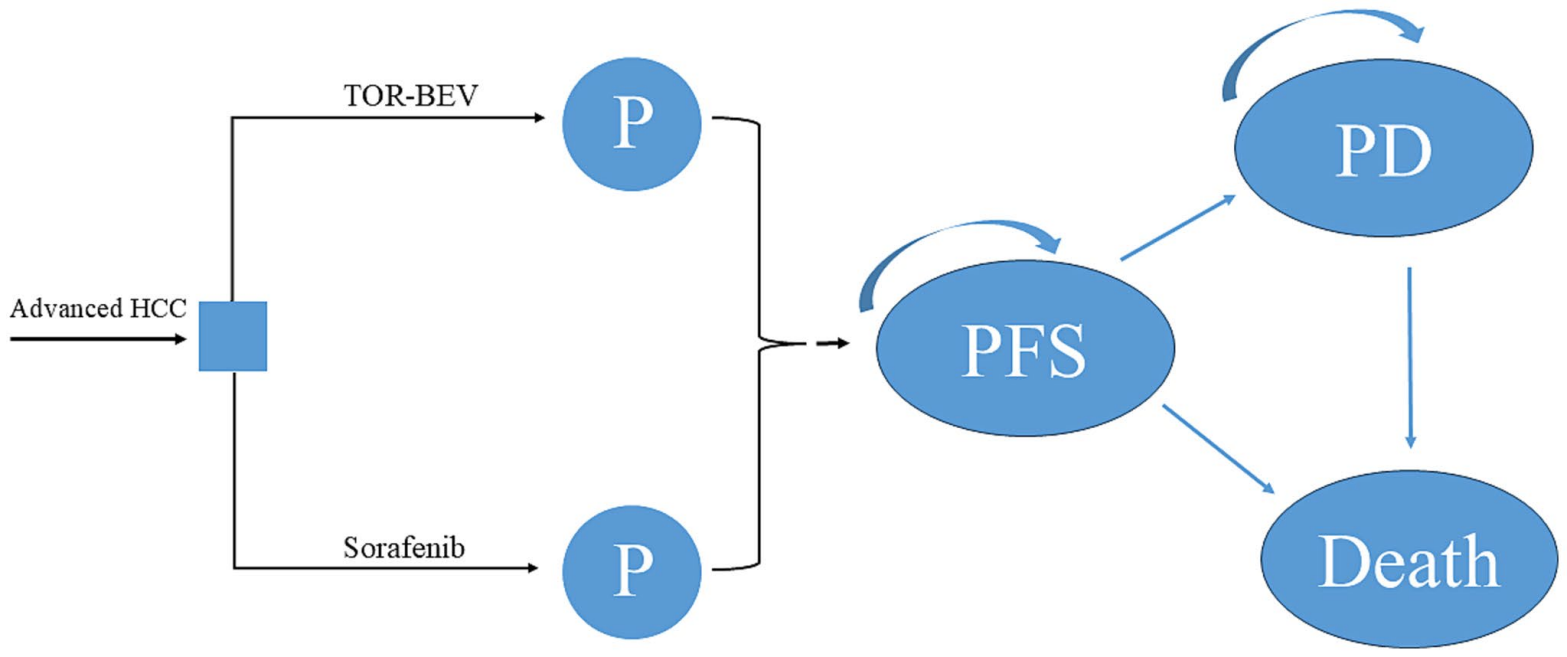
The partitioned survival model simulating outcomes for the HEPATORCH trial. All patients started with PFS state and received treatment with TOR-BEV or sorafenib. HCC, hepatocellular carcinoma; PD, disease progression; PFS, progression-free survival; TOR-BEV, toripalimab plus bevacizumab.

A partitioned survival model was constructed using TreeAge 2022 software to evaluate the cost-effectiveness of TOR-BEV and sorafenib as first-line treatments for advanced HCC (Figure 1). The model consists of three mutually exclusive health states: PFS, disease progression (PD), and death. All patients are assumed to begin in the PFS state. As the model runs, patients either remain in their current state or transition to the next one, but cannot revert to previous states (16). Each cycle of the model corresponds to 21 days, based on the medication cycle from the HEPATORCH trial. The model simulates 205 cycles, approximately 11.8 years, by which time nearly 95% of patients are expected to have died. The key outcomes include total costs, quality-adjusted life-years (QALYs), and incremental cost-effectiveness ratios (ICERs). The willingness-to-pay (WTP) threshold was set at $40,365 per QALY, which is three times the per-capita GDP of China in 2024 (17). A treatment is deemed cost-effective if its ICER is below this threshold.

### 2.2 Study population

The treatment and efficacy data for this study were sourced from the HEPATORCH trial (12), a phase III multicenter randomized controlled trial conducted across 57 hospitals in mainland China, Taiwan, and Singapore. Eligibility criteria for the trial included patients aged 18 to 75 years, with histologically or cytologically confirmed untreated HCC, not amenable to radical or locoregional therapies. Eligible patients were randomized to receive either TOR-BEV or sorafenib. In the TOR-BEV group, the treatment regimen is administered in 21-day cycles. Toripalimab is administered intravenously at a dose of 240 mg once per cycle, and bevacizumab is administered intravenously at a dose of 15 mg/kg once per cycle. In the sorafenib group, patients received 400 mg of oral sorafenib twice daily, with each cycle lasting three weeks. Treatment continued until PD, intolerance, or completion of 35 cycles of toripalimab. In the model, we assumed that the duration of first-line pharmacotherapy equals the median treatment exposure observed in the HEPATORCH trial—29.9 weeks for toripalimab, 26.6 weeks for bevacizumab, and 15.1 weeks for sorafenib. However, due to the lack of detailed data on post-progression treatment, this study assumes that some patients received regorafenib as second-line therapy, while others received the best supportive care.

### 2.3 State membership

To extract survival data, GetData Graph Digitizer software (version 2.26) was used to digitize the Kaplan–Meier survival curves from the HEPATORCH trial. Following the methodology of Guyot et al. (18), R software was employed to reconstruct patient survival curves from the digitized data points. These curves were fitted to various survival distributions, including exponential, gamma, generalized F, generalized gamma, Gompertz, Weibull, log-logistic, and log-normal (19, 20). Based on the Akaike and Bayesian information criteria (21), the log-logistic distribution was identified as the best fit for the PFS and OS curves in both the TOR-BEV and sorafenib groups (Supplementary Table 2). These distributions were used to calculate state memberships between health states (Supplementary Figure 1), with specific parameters of the log-logistic distribution presented in Table 1.

**Table 1:** Relevant parameters of survival distribution.

Variable	Value	Source
Log-logistic survival model of PFS		
TOR-BEV group	Scale = 0.1778130, Shape = 1.475571	(12)
Sorafenib group	Scale = 0.2730645, Shape = 1.880531	(12)
Log-logistic survival model of OS		
TOR-BEV group	Scale = 0.05323438, Shape = 1.454756	(12)
Sorafenib group	Scale = 0.06767017, Shape = 1.602551	(12)

OS, overall survival; PFS, progression-free survival; TOR-BEV, toripalimab plus bevacizumab.

### 2.4 Costs and utilities

This study focused on direct medical costs, including drugs, tests, routine follow-up, management of grade 3 or higher adverse events with an incidence rate of 5% or more, best supportive care, and end-of-life care (Table 2). Drug costs were derived from national bidding prices, while other cost data were extracted from publicly available literature and adjusted to 2024 levels using China’s medical price index from the National Bureau of Statistics (22). All costs were converted to US dollars using the 2024 average exchange rate of 7.12 CNY per USD. Utility values for quality-of-life in each health state range from 0 to 1, where 1 represents perfect health and 0 represents death. Since the HEPATORCH trial (12) did not provide quality-of-life data, this study used utility values for PFS and PD from Chinese studies (23). The disutility associated with grade 3 or higher adverse events, occurring in 5% or more of patients, was also incorporated to minimize potential bias. All costs and utilities were discounted at an annual rate of 5% (17). Drug dose calculations were based on a standard patient weight of 65 kg (Table 2) (24).

**Table 2:** Basic parameters of the input model and the range of sensitivity analyses.

Variable	Base Value	Range		Distribution	Source
		Min	Max		
Risk of AEs					
TOR-BEV group: incidence of AEs					
Thrombocytopenia	0.062	0.050	0.074	Beta	(12)
Hypertension	0.160	0.128	0.192	Beta	(12)
Aspartate aminotransferase increased	0.031	0.025	0.037	Beta	(12)
Anaemia	0.056	0.045	0.067	Beta	(12)
Diarrhoea	0.012	0.010	0.014	Beta	(12)
Hepatic function abnormal	0.056	0.045	0.067	Beta	(12)
Palmar-plantar erythrodysesthesia syndrome	0	NA	NA	NA	
Sorafenib group: incidence of AEs					
Thrombocytopenia	0.024	0.019	0.029	Beta	(12)
Hypertension	0.116	0.093	0.139	Beta	(12)
Aspartate aminotransferase increased	0.055	0.044	0.066	Beta	(12)
Anaemia	0.043	0.034	0.052	Beta	(12)
Diarrhoea	0.073	0.058	0.088	Beta	(12)
Hepatic function abnormal	0.030	0.024	0.036	Beta	(12)
Palmar-plantar erythrodysaesthesia syndrome	0.104	0.083	0.125	Beta	(12)
Cost ($)					
Toripalimab (240 mg)	264.73	211.78	317.68	Gamma	(25)
Bevacizumab (100 mg)	134.41	107.53	161.29	Gamma	(25)
Sorafenib (200 mg)	1.91	1.53	2.29	Gamma	(25)
Regorafenib (40 mg)	1.20	0.96	1.44	Gamma	(25)
Thrombocytopenia	322.79	258.23	387.35	Gamma	(26)
Hypertension	155.87	124.70	187.04	Gamma	(26)
Aspartate aminotransferase increased	182.21	145.77	218.65	Gamma	(23)
Anaemia	336.97	269.58	404.36	Gamma	(27)
Diarrhoea	28.78	23.02	34.54	Gamma	(28)
Hepatic function abnormal	182.21	145.77	218.65	Gamma	(23)
Palmar-plantar erythrodysaesthesia syndrome	16.66	13.33	19.99	Gamma	(26)
Best supportive care per cycle	182.59	146.07	219.11	Gamma	(29)
Routine follow-up per cycle	73.79	59.03	88.55	Gamma	(29)
Test per cycle	357.70	286.16	429.24	Gamma	(30)
Terminal care in end-of-life	1,491	1,192.80	1,789.20	Gamma	(20)
Utility values					
PFS	0.76	0.61	0.91	Beta	(23)
PD	0.68	0.54	0.82	Beta	(23)
Disutility of AEs					
Thrombocytopenia	0.140	0.112	0.168	Beta	(31)
Hypertension	0.016	0.013	0.019	Beta	(32)
Aspartate aminotransferase increased	0	NA	NA	NA	(33)
Anaemia	0.070	0.056	0.084	Beta	(34)
Diarrhoea	0.047	0.038	0.056	Beta	(35)
Hepatic function abnormal	0	NA	NA	NA	(33)
Palmar-plantar erythrodysaesthesia syndrome	0.15	0.12	0.18	Beta	(32)
Discount rate	0.05	0	0.08	Fixed	(17)
Weight (Kg)	65	52	78	Normal	(24)
The proportion of subsequent anticancer therapies					
TOR-BEV group					
Regorafenib	0.475	0.380	0.570	Beta	(12)
Sorafenib group					
Regorafenib	0.579	0.463	0.695	Beta	(12)

AE, adverse event; PD, disease progression; PFS, progression-free survival; TOR-BEV, toripalimab plus bevacizumab.

### 2.5 Sensitivity analysis

A sensitivity analysis was conducted to evaluate the robustness of the model’s results, including both one-way and probabilistic sensitivity analyses. The one-way sensitivity analysis was designed to assess the impact of varying individual parameters within specific ranges on the cost-effectiveness outcomes. Each parameter was adjusted within the 95% confidence interval reported in the literature. In cases where the data was insufficient, the base case value was modified by ±20%. Consistent with the China Guidelines for Pharmacoeconomic Evaluation, the discount rate was tested within a range of 0 to 8% (Table 2) (17). The results of this analysis are presented in a tornado diagram, providing a clear visual representation of the impact of each parameter on the ICER. To further explore the uncertainty in the cost-effectiveness results, a probabilistic sensitivity analysis was performed. For this, each parameter was assigned a specific distribution (Table 2), and a Monte Carlo simulation with 1,000 iterations was conducted. In each iteration, random samples were drawn from the input distributions. The results are presented *via* a cost-effectiveness acceptability curve and a scatter plot, which visually depict the probability of the two treatment regimens being cost-effective at varying WTP thresholds. Additionally, we repeated the calculation of ICER values in the TOR-BEV group compared to the sorafenib group by continuously reducing the price of toripalimab and bevacizumab to explore the corresponding price of toripalimab and bevacizumab when TOR-BEV is cost-effective.

### 2.6 Subgroup analysis

To assess the uncertainty in cost-effectiveness results arising from variations in baseline characteristics across different subgroups, an exploratory subgroup analysis was conducted (Table 3). This analysis focused on prespecified subgroups from the HEPATORCH trial, using subgroup-specific hazard ratios for OS and PFS provided by the trial, and applied the method outlined by Hoyle et al. (36). We assumed that, for the sorafenib group, all subgroup-specific PFS and OS curves coincide with those of the overall population (shape and scale parameters unchanged). For the TOR-BEV group, the shape parameter of each subgroup was set equal to that of the corresponding sorafenib stratum, while the scale parameter was obtained by multiplying the sorafenib scale by the reported hazard ratio.

**Table 3:** Results of subgroup analyses.

Subgroup	PFS HR (95% CI)	OS HR (95% CI)	ICER ($/QALY)	Cost-effectiveness probability (%)
Age, years				
<65	0.76 (0.55–1.03)	0.85 (0.63–1.14)	93758.28	15.3
≥65	0.38 (0.21–0.72)	0.54 (0.31–0.95)	44647.69	34.7
Sex				
Male	0.62 (0.47–0.84)	0.76 (0.57–1.00)	66800.17	1.8
Female	0.91 (0.42–2.07)	0.81 (0.40–1.67)	148363.98	0
ECOG				
0	0.65 (0.43–0.99)	0.89 (0.62–1.27)	139879.34	0
1	0.70 (0.49–0.99)	0.63 (0.43–0.92)	37532.39	63.2
Alpha-fetoprotein concentration, ng/mL				
<400	0.57 (0.38–0.83)	0.71 (0.48–1.04)	57822.15	8.9
≥400	0.81 (0.55–1.20)	0.79 (0.56–1.13)	63250.77	3.6
BCLC stage				
B	0.78 (0.43–1.40)	0.69 (0.38–1.23)	42503.53	45.2
C	0.66 (0.48–0.89)	0.80 (0.60–1.07)	77301.61	0.3
Macrovascular invasion or extrahepatic spread				
Yes	0.66 (0.48–0.89)	0.80 (0.60–1.07)	77301.61	0.3
No	0.78 (0·43–1·40)	0.69 (0.38–1.23)	42503.53	43.9
Macrovascular invasion				
Yes	0.70 (0.43–1.13)	0.73 (0.48–1.11)	53751.31	13.4
No	0.66 (0.47–0.92)	0.77 (0.55–1.08)	66772.82	1.0
Extrahepatic spread				
Yes	0.61 (0.42–0.90)	0.86 (0.59–1.24)	116192.59	0
No	0.75 (0.51–1.11)	0.70 (0.48–1.01)	45410.03	36.7
Hepatitis B virus aetiology				
Yes	0.67 (0.50–0.89)	0.77 (0.59–1.01)	66041.21	2.1
No	0.74 (0.29–1.84)	0.87 (0.37–2.02)	110030.8	0
Previous local therapy				
Yes	0.63 (0.43–0.93)	0.81 (0.53–1.22)	84218.26	0.2
No	0.70 (0.47–1.03)	0.69 (0.49–0.97)	46065.34	31.1

BCLC, Barcelona clinic liver cancer; CI, confidence interval; ECOG, eastern cooperative oncology group HR, hazard ratio; ICER, incremental cost-effectiveness ratio; OS, overall survival; PFS, progression-free survival.

### 2.7 Scenario analysis

Two scenario analyses were performed. In Scenario 1, considering the unique long-tail effect of immunotherapy in cancer treatment, the model duration was adjusted to 15 and 20 years. This analysis aimed to assess how different time horizons affect the cost-effectiveness results. In Scenario 2, recognizing the economic diversity and varying healthcare resource availability across China, the WTP threshold was adjusted to three times the per capita GDP for Beijing, Inner Mongolia, and Gansu. These regions represent high, moderate, and low economic development areas in China, respectively, and were used to evaluate the cost-effectiveness of TOR-BEV under different economic contexts.

## 3 Results

### 3.1 Base case analysis

The TOR-BEV group incurred total costs of $42,239.26 and yielded 1.88 QALYs, whereas the sorafenib group had total costs of $11,201.54 and produced 1.43 QALYs. The ICER for the TOR-BEV group compared to the sorafenib group was $69,231.90 per QALY (Table 4). Since this ICER exceeds the predefined WTP threshold of $40,365 per QALY, TOR-BEV is not deemed cost-effective as a first-line treatment for advanced HCC from the perspective of China’s healthcare system when compared to sorafenib.

**Table 4:** The cost and outcome results of the cost-effectiveness analysis.

Regimen	TOR-BEV group	Sorafenib group	Incremental
Total QALYs	1.88	1.43	0.45
Total cost, $	42,239.26	11,201.54	31,037.71
ICER, $ Per QALY			69,231.90

ICER, incremental cost-effectiveness ratio; QALY, quality-adjusted life year; TOR-BEV, toripalimab plus bevacizumab.

### 3.2 Sensitivity analysis

**Figure 2:**
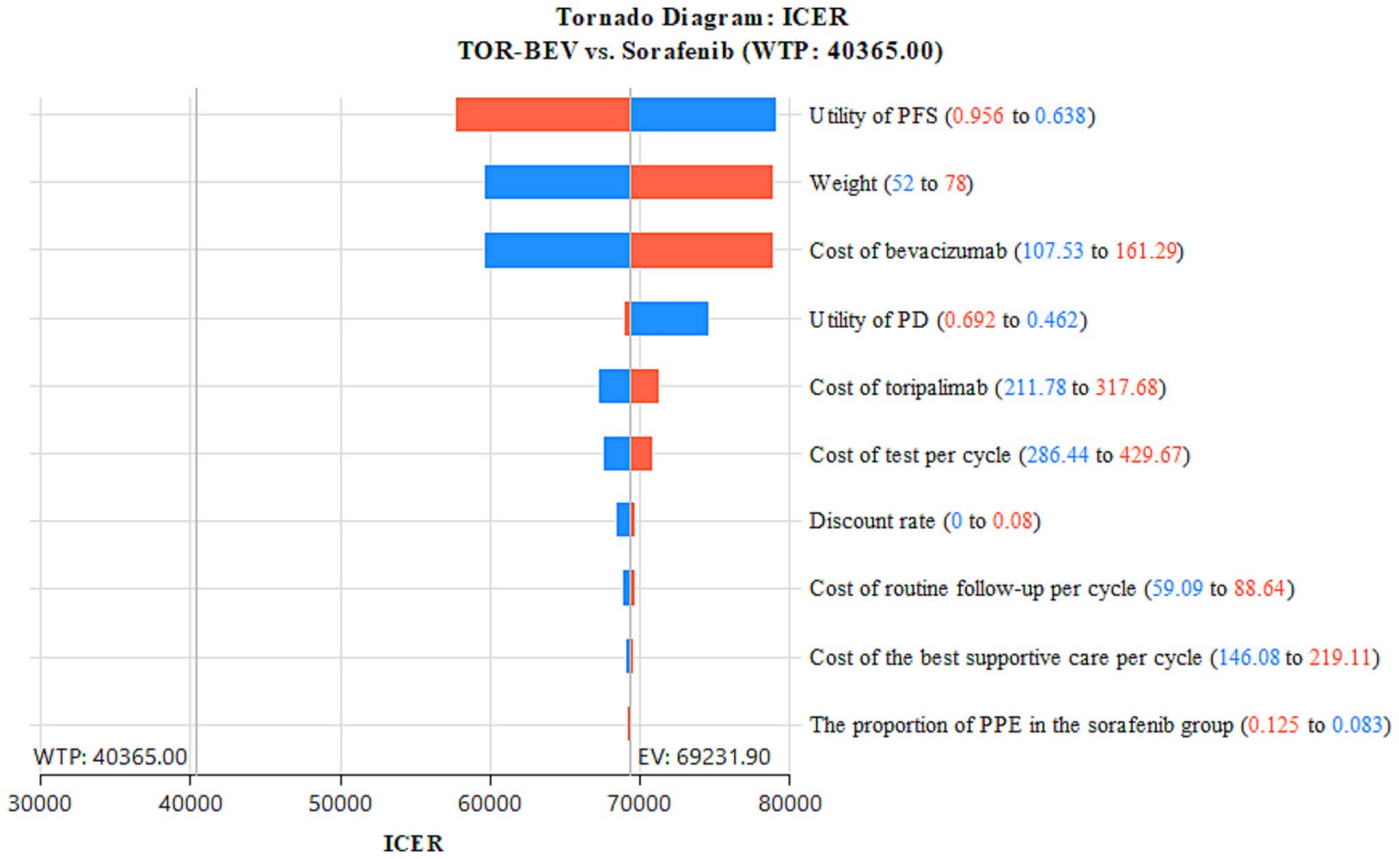
One-way sensitivity analyses of TOR-BEV in comparison to sorafenib. ICER, incremental cost-effectiveness ratio; PD, disease progression; PFS, progression-free survival; PPE, palmar-plantar erythrodysaesthesia syndrome; TOR-BEV, toripalimab plus bevacizumab; WTP, willingness-to-pay.

**Figure 3:**
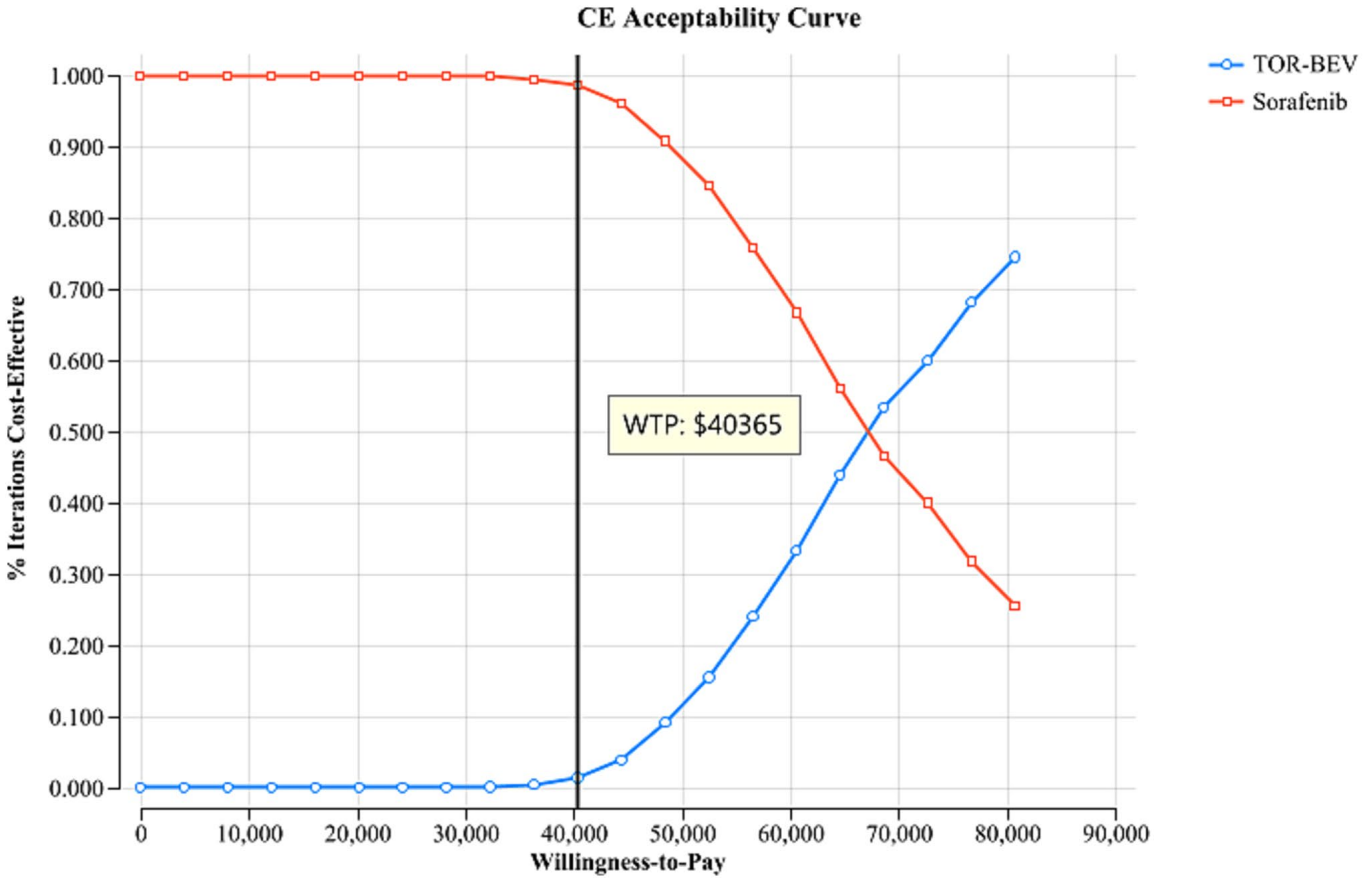
The cost-effectiveness acceptability curve illustrates the probability that one treatment option is cost-effective compared to another at various WTP thresholds. CE, cost-effectiveness; TOR-BEV, toripalimab plus bevacizumab; WTP, willingness-to-pay.

**Figure 4:**
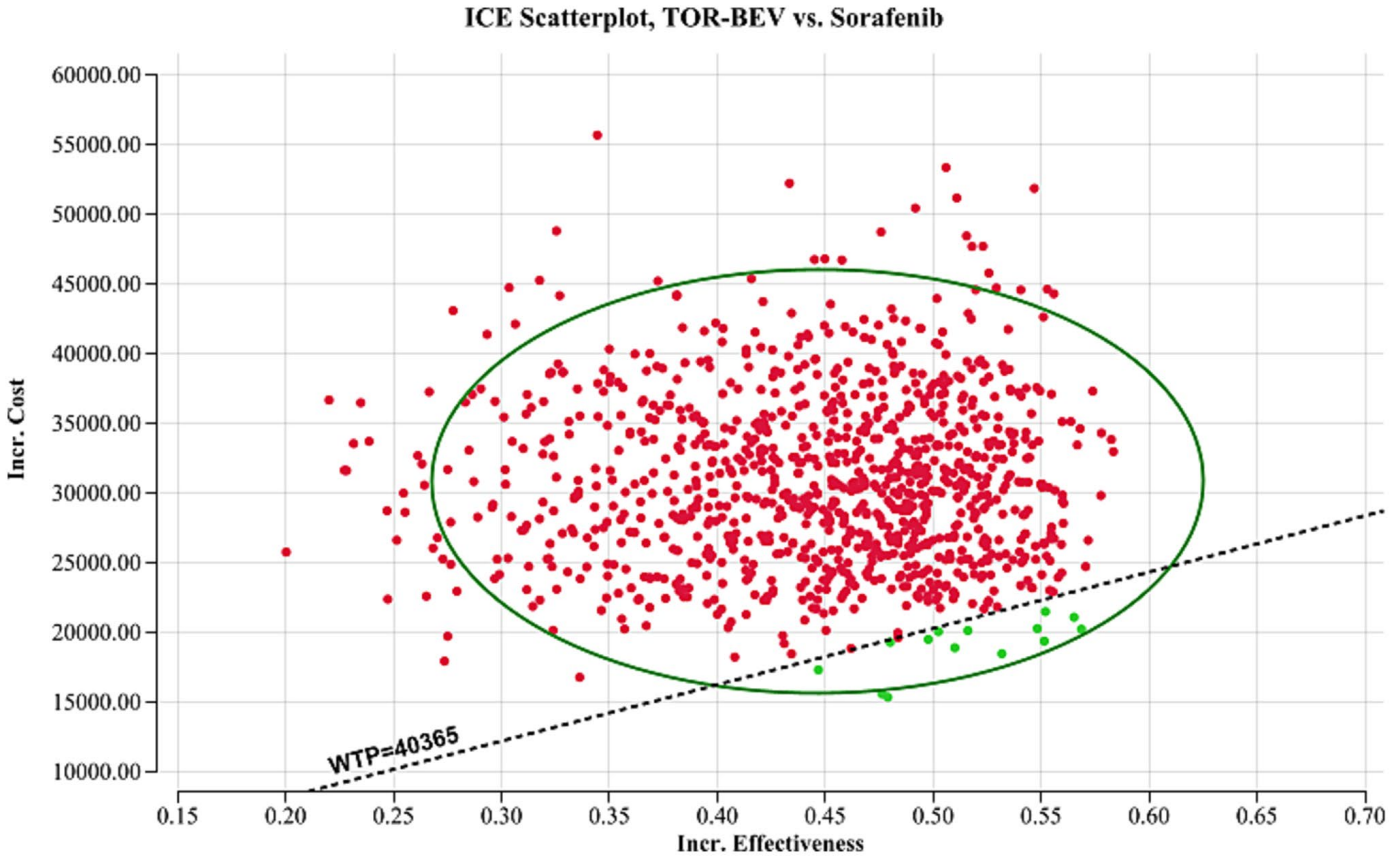
A probabilistic scatter plot of the ICER between the TOR-BEV and sorafenib. Each point means the ICER for 1 simulation. Ellipses are used to indicate 95% confidence intervals. Points that lie below the ICER threshold represent cost-effective simulations. ICE, incremental cost-effectiveness; TOR-BEV, toripalimab plus bevacizumab; WTP, willingness-to-pay.

The one-way sensitivity analysis, represented in the tornado diagram (Figure 2), identified key parameters, such as PFS utility value, weight, cost of bevacizumab, PD utility value, and cost of toripalimab, as having significant impacts on the model. However, even when these parameters were varied within their specified ranges, the ICER remained above the established WTP threshold, indicating their limited effect on the model’s outcome. Other parameters had relatively smaller impacts. The probabilistic sensitivity analysis results, shown in the cost-effectiveness acceptability curve (Figure 3) and scatter plot (Figure 4), revealed that when the WTP threshold is set at $40,365 per QALY, the probability of TOR-BEV being cost-effective compared to sorafenib is only 1.5%. TOR-BEV would only become cost-effective if the prices of toripalimab and bevacizumab were reduced by more than 50.1%, bringing their costs down to $132.63 and $67.34, respectively.

### 3.3 Subgroup analysis

The subgroup analysis results, presented in Table 3, show that only the Eastern Cooperative Oncology Group performance status score of 1 subgroup demonstrated an ICER below the predefined WTP threshold, indicating cost-effectiveness. Other subgroups did not meet the cost-effectiveness criteria. Notably, although the Barcelona Clinic Liver Cancer stage B and no macrovascular invasion or extrahepatic spread subgroups did not demonstrate cost-effectiveness, their ICERs were relatively close to the predefined WTP threshold.

### 3.4 Scenario analysis

The results of the Scenario 1 analysis, outlined in Table 5, indicated that when the model duration was adjusted to 15 and 20 years, TOR-BEV remained non-costeffective compared to sorafenib. However, as the model duration increased, the ICER for TOR-BEV decreased, and its probability of being cost-effective gradually rose. In Scenario 2, with WTP thresholds set at three times the per capita GDP of Beijing, Inner Mongolia, and Gansu ($96,072/QALY, $46,275/QALY, and $22,224/QALY, respectively), the probability of TOR-BEV being cost-effective compared to sorafenib was 88.7, 4.9, and 0%, respectively. This suggests that TOR-BEV could be considered cost-effective when the WTP threshold corresponds to three times Beijing’s per capita GDP.

**Table 5:** Results of scenario analyses.

Scenario	Cost ($)		QALY		ICER ($/QALY)
	TOR-BEV group	Sorafenib group	TOR-BEV group	Sorafenib group	
Scenario 1					
Model runtime (year) = 15	43,404.35	11,478.74	1.96	1.47	65,047.01
Model runtime (year) = 20	44,652.06	11765.14	2.05	1.51	61,054.63

ICER, incremental cost-effectiveness ratio; QALY, quality-adjusted life year; TOR-BEV, toripalimab plus bevacizumab.

## 4 Discussion

To our knowledge, this study is the first to assess and compare the cost-effectiveness of TOR-BEV and sorafenib as first-line treatments for advanced HCC from the perspective of China’s healthcare system, based on the latest clinical evidence. This represents a key innovation of the study. Our analysis shows that compared to sorafenib, TOR-BEV increases QALYs by 0.45, with an additional cost of $31,037.71, resulting in an ICER of $69,231.90 per QALY, which exceeds the predefined WTP threshold. As a result, from the perspective of China’s healthcare system, TOR-BEV is not considered cost-effective when compared to sorafenib. This may be due to several challenges faced by novel anti-tumor drugs, including lengthy development timelines, high costs, low success rates, and patent protections, all of which contribute to high market prices (37). Consequently, the costs of toripalimab and bevacizumab are significantly higher than sorafenib, making the treatment cost per cycle for the TOR-BEV regimen more expensive without delivering sufficient incremental survival benefits. The one-way sensitivity analysis highlights that, aside from utility values, key factors influencing the model’s outcomes include patient weight (used to calculate the bevacizumab dose) and the costs of bevacizumab and toripalimab, which supports the previous conclusion. Since its establishment in 2018, China’s National Healthcare Security Administration has been actively promoting national procurement strategies and conducting multiple rounds of price negotiations with pharmaceutical companies. These efforts aim to improve the accessibility of anti-cancer drugs and significantly reduce the financial burden on society and cancer patients. As a result, the prices of many anti-cancer drugs have dropped significantly, with reductions ranging from 30 to 70% (38). Given this, the potential price reductions of toripalimab and bevacizumab that would make TOR-BEV cost-effective were explored. These findings suggest that TOR-BEV could become cost-effective if the prices of both drugs were reduced to below 50.1% of their current prices. This provides valuable economic insights for future price negotiations and medical insurance discussions for these drugs. This threshold provides valuable economic insights for future price negotiations, particularly for the National Healthcare Security Administration. Notably, it serves as a concrete, evidence-based target in drug reimbursement decision-making, facilitating sustainable access to innovative oncology therapies while ensuring healthcare fund affordability.

One-way sensitivity analysis indicates that the model outcomes remain stable when the values of model parameters are varied within a specified range. The probabilistic sensitivity analysis further demonstrates that, when used as a first-line treatment for advanced HCC, the probability of TOR-BEV being cost-effective compared to sorafenib is only 1.5%. These findings provide evidence of the model’s robustness from different analytical perspectives. The Scenario 1 analysis reveals that as the model duration increases, the ICER for TOR-BEV compared to sorafenib decreases, suggesting that TOR-BEV becomes more cost-effective with longer treatment durations. This trend may improve patient adherence to long-term treatment, thus having a positive ethical impact. Scenario 2 highlights that TOR-BEV is cost-effective in economically advanced regions, such as Beijing, but not in moderately developed areas like Inner Mongolia or underdeveloped regions like Gansu. This underscores the importance of considering local economic conditions and the availability of medical resources in drug policy and resource allocation decisions, aiming to achieve cost-effective distribution, promote medical equity, and enhance the overall quality and efficiency of healthcare services. Subgroup analysis suggests that only the Eastern Cooperative Oncology Group performance status score of 1 subgroup is cost-effective, while the Barcelona Clinic Liver Cancer stage B and no macrovascular invasion or extrahepatic spread subgroups are nearly cost-effective. This suggests that personalized treatment plans, tailored to individual patient characteristics, could optimize cost-effectiveness, providing valuable guidance for healthcare policymakers in establishing appropriate reimbursement criteria. To the best of our knowledge, several pharmacoeconomic analyses of ICIs combined with bevacizumab for advanced HCC treatment have been conducted, primarily focusing on atezolizumab and sintilimab. Studies by Hou et al. (39), Wen et al. (40), Su et al. (19), and Liu et al. (24) concluded that atezolizumab plus bevacizumab is unlikely to be a cost-effective first-line option for patients with advanced HCC compared to sorafenib, which aligns with our findings. In contrast, Ye et al. (41) and Zhou et al. (42) considered sintilimab combined with bevacizumab biosimilars a cost-effective first-line therapy for advanced HCC, contradicting our results. The choice of comparator is a critical step in cost-effectiveness analysis. According to the 2024 Chinese Clinical Guidelines for Primary Liver Cancer, first-line systemic therapies for advanced HCC include—not only sorafenib—but also regimens such as atezolizumab plus bevacizumab and sintilimab plus bevacizumab. However, no head-to-head trial data are available comparing TOR-BEV with these immune-combination regimens. Therefore, we selected sorafenib as the comparator, which has direct phase III evidence (HEPATORCH trial) and is concurrently recommended by the guidelines, to ensure consistent efficacy inputs and to keep uncertainty manageable.

It should be noted that this study employs a partitioned survival model based on the following assumptions: patients are partitioned into three mutually exclusive and irreversible health states (PFS, PD, and dead); the state proportions are derived from independently fitted PFS and OS curves; and the relative treatment effect observed during follow-up is assumed to remain constant over the extrapolation period. These assumptions impose inherent limitations: the model cannot capture repeated progression or backward transitions; the absence of structural constraints between PFS and OS may produce structural inconsistency (i.e., the proportion alive and progression-free exceeding the proportion alive) if extrapolation is misspecified; and long-term survival probabilities are highly sensitive to the chosen parametric functional form. Insufficient follow-up coupled with minor mis-specification can substantially inflate the uncertainty in cost-effectiveness analysis results and may alter the cost-effectiveness conclusion.

This study has several key strengths. First, it incorporates the latest HEPATORCH trial data, which directly compares the efficacy of TOR-BEV and sorafenib, providing cutting-edge cost-effectiveness evidence. Second, as all participants in the HEPATORCH trial are Asian patients, with 96.3% from mainland China, the trial’s results are highly applicable to cost-effectiveness analyses in the Chinese population. Finally, the detailed subgroup and scenario analyses offer valuable economic insights for various patient groups and treatment settings, serving as an important resource for physicians, patients, and policymakers when selecting personalized treatment plans and making informed decisions.

However, this study has several limitations. First, to simplify the model, only grade 3 or higher adverse reactions with an incidence rate of ≥5% were included, which may underestimate treatment costs. Nonetheless, the one-way sensitivity analysis indicates that adverse reaction management costs do not significantly affect the model’s results. Second, the HEPATORCH trial does not provide quality-of-life data, so survival utility values from Chinese literature were used, which may introduce bias. However, one-way sensitivity analysis suggests this does not alter the model’s outcomes. Third, due to the ongoing nature of the HEPATORCH trial, long-term survival data are unavailable. Extrapolation of survival data beyond the follow-up period using survival models may not fully reflect actual outcomes. The study will be updated once long-term data are available. Fourth, the HEPATORCH trial does not provide detailed information on treatments after PD. The assumption that patients receive regorafenib or best supportive care as second-line treatment may not fully capture real-world clinical practices. Fifth, the subgroup analysis used subgroup-specific hazard ratios to proportionally shift the survival curves, assuming time-invariant treatment effects. If the true efficacy declines over time, this assumption will over-extrapolate tail survival and may systematically inflate lifetime QALYs and ICERs. Coupled with limited sample sizes in each subgroup, these factors warrant cautious interpretation of the subgroup findings. Despite these limitations, the study offers valuable economic insights for policymakers, healthcare providers, and patients.

## 5 Conclusion

From the perspective of the Chinese healthcare system, TOR-BEV is unlikely to be cost-effective as a first-line treatment for advanced HCC compared to sorafenib unless the prices of both toripalimab and bevacizumab are reduced to below 50.1% of their current prices. However, this regimen was cost-effective in the subgroup of patients with an Eastern Cooperative Oncology Group performance status score of 1, and economic viability is also expected in highly developed regions such as Beijing.

## Data Availability

The original contributions presented in the study are included in the article/Supplementary material, further inquiries can be directed to the corresponding author.
