# Supplementary material for: Cost-effectiveness analysis of toripalimab plus bevacizumab as first-line therapy for advanced hepatocellular carcinoma
Source: Front Public Health. 2026 Mar 30;14:1649775. doi: 10.3389/fpubh.2026.1649775 (PMC13070910; doi:10.3389/fpubh.2026.1649775)
Supplement: Supplementary file 1 [file Data_Sheet_1.docx]

Supplementary Material

**Cost-Effectiveness Analysis of Toripalimab Plus Bevacizumab as First-line Therapy for Advanced Hepatocellular Carcinoma**

1. Supplementary Table 1. CHEERS 2022 Checklist.

2. Supplementary Table 2. Comparison of survival models.

3. Supplementary Figure 1. Results of the survival curve fit the TOR-BEV group and sorafenib group.

1. Supplementary Table 1. CHEERS 2022 Checklist.

| **Topic** | **No.** | **Item** | **Reported** |
| --- | --- | --- | --- |
| **Title** |  |  |  |
|  | 1 | Identify the study as an economic evaluation and specify the interventions being compared. | Title |
| **Abstract** |  |  |  |
|  | 2 | Provide a structured summary that highlights context, key methods, results, and alternative analyses. | Abstract |
| **Introduction** |  |  |  |
| **Background and objectives** | 3 | Give the context for the study, the study question, and its practical relevance for decision making in policy or practice. | Introduction |
| **Methods** |  |  |  |
| **Health economic analysis plan** | 4 | Indicate whether a health economic analysis plan was developed and where available. | Introduction |
| **Study population** | 5 | Describe characteristics of the study population (such as age range, demographics, socioeconomic, or clinical characteristics). | 2.2 Study Population |
| **Setting and location** | 6 | Provide relevant contextual information that may influence findings. | 2.2 Study Population |
| **Comparators** | 7 | Describe the interventions or strategies being compared and why chosen. | Discussion |
| **Perspective** | 8 | State the perspective(s) adopted by the study and why chosen. | Introduction |
| **Time horizon** | 9 | State the time horizon for the study and why appropriate. | 2.1 Model Construction |
| **Discount rate** | 10 | Report the discount rate(s) and reason chosen. | 2.4 Costs and Utilities |
| **Selection of outcomes** | 11 | Describe what outcomes were used as the measure(s) of benefit(s) and harm(s). | 2.1 Model Construction |
| **Measurement of outcomes** | 12 | Describe how outcomes used to capture benefit(s) and harm(s) were measured. | 2.1 Model Construction |
| **Valuation of outcomes** | 13 | Describe the population and methods used to measure and value outcomes. | 2.1 Model Construction |
| **Measurement and valuation of resources and costs** | 14 | Describe how costs were valued. | 2.4 Costs and Utilities |
| **Currency, price date, and conversion** | 15 | Report the dates of the estimated resource quantities and unit costs, plus the currency and year of conversion. | 2.4 Costs and Utilities |
| **Rationale and description of model** | 16 | If modelling is used, describe in detail and why used. Report if the model is publicly available and where it can be accessed. | 2.1 Model Construction, Discussion |
| **Analytics and assumptions** | 17 | Describe any methods for analysing or statistically transforming data, any extrapolation methods, and approaches for validating any model used. | 2.3 State Membership |
| **Characterising heterogeneity** | 18 | Describe any methods used for estimating how the results of the study vary for subgroups. | 2.6 Subgroup Analysis |
| **Characterising distributional effects** | 19 | Describe how impacts are distributed across different individuals or adjustments made to reflect priority populations. | 2.5 Sensitivity Analysis |
| **Characterising uncertainty** | 20 | Describe methods to characterise any sources of uncertainty in the analysis. | 2.5 Sensitivity Analysis |
| **Approach to engagement with patients and others affected by the study** | 21 | Describe any approaches to engage patients or service recipients, the general public, communities, or stakeholders (such as clinicians or payers) in the design of the study. | Not applicable |
| **Results** |  |  |  |
| **Study parameters** | 22 | Report all analytic inputs (such as values, ranges, references) including uncertainty or distributional assumptions. | Table 2 |
| **Summary of main results** | 23 | Report the mean values for the main categories of costs and outcomes of interest and summarise them in the most appropriate overall measure. | Table 4 |
| **Effect of uncertainty** | 24 | Describe how uncertainty about analytic judgments, inputs, or projections affect findings. Report the effect of choice of discount rate and time horizon, if applicable. | 3.2 Sensitivity Analysis |
| **Effect of engagement with patients and others affected by the study** | 25 | Report on any difference patient/service recipient, general public, community, or stakeholder involvement made to the approach or findings of the study | Not applicable |
| **Discussion** |  |  |  |
| **Study findings, limitations, generalisability, and current knowledge** | 26 | Report key findings, limitations, ethical or equity considerations not captured, and how these could affect patients, policy, or practice. | Discussion |
| **Other relevant information** |  |  |  |
| **Source of funding** | 27 | Describe how the study was funded and any role of the funder in the identification, design, conduct, and reporting of the analysis | Funding |
| **Conflicts of interest** | 28 | Report authors conflicts of interest according to journal or International Committee of Medical Journal Editors requirements. | Declaration of Conflicting Interests |

2. Supplementary Table 2. Comparison of survival models.

|  | AIC | | BIC | |
| --- | --- | --- | --- | --- |
|  | TOR-BEV group | sorafenib group | TOR-BEV group | sorafenib group |
| OS | | |  |  |
| Exponential | 918.4885 | 1048.662 | 921.5761 | 1051.762 |
| Gamma | 916.7912 | 1043.764 | 922.9664 | 1049.963 |
| Gen.F | 902.7304 | 1043.787 | 915.0808 | 1056.187 |
| Gen.Gamma | 900.7222 | 1041.774 | 909.9850 | 1051.073 |
| Gompertz | 920.4874 | 1049.167 | 926.6626 | 1055.367 |
| Weibull | 918.3540 | 1045.382 | 924.5292 | 1051.582 |
| Log-logistic | 901.2268 | 1039.678 | 907.4020 | 1047.878 |
| Log-normal | 904.7486 | 1040.269 | 910.9238 | 1046.469 |
| PFS | |  |  |  |
| Exponential | 611.2385 | 622.2580 | 614.2845 | 625.3579 |
| Gamma | 602.6633 | 609.2380 | 604.5214 | 615.4378 |
| Gen.F | 599.6825 | 603.9567 | 557.8452 | 566.3562 |
| Gen.Gamma | 554.8572 | 564.5660 | 563.8547 | 573.8656 |
| Gompertz | 612.8418 | 624.1588 | 619.5689 | 630.3585 |
| Weibull | 606.8544 | 615.6059 | 611.5477 | 621.8056 |
| Log-logistic | 582.2451 | 593.6382 | 559.2158 | 559.8379 |
| Log-normal | 585.5148 | 596.8487 | 584.5641 | 593.0484 |

AIC, Akaike information criterion; BIC, Bayesian Information Criterion; OS, overall survival; PFS, progression-free survival; TOR-BEV, toripalimab plus bevacizumab.

3. Supplementary Figure 1. Results of the survival curve fit the TOR-BEV group and sorafenib group.


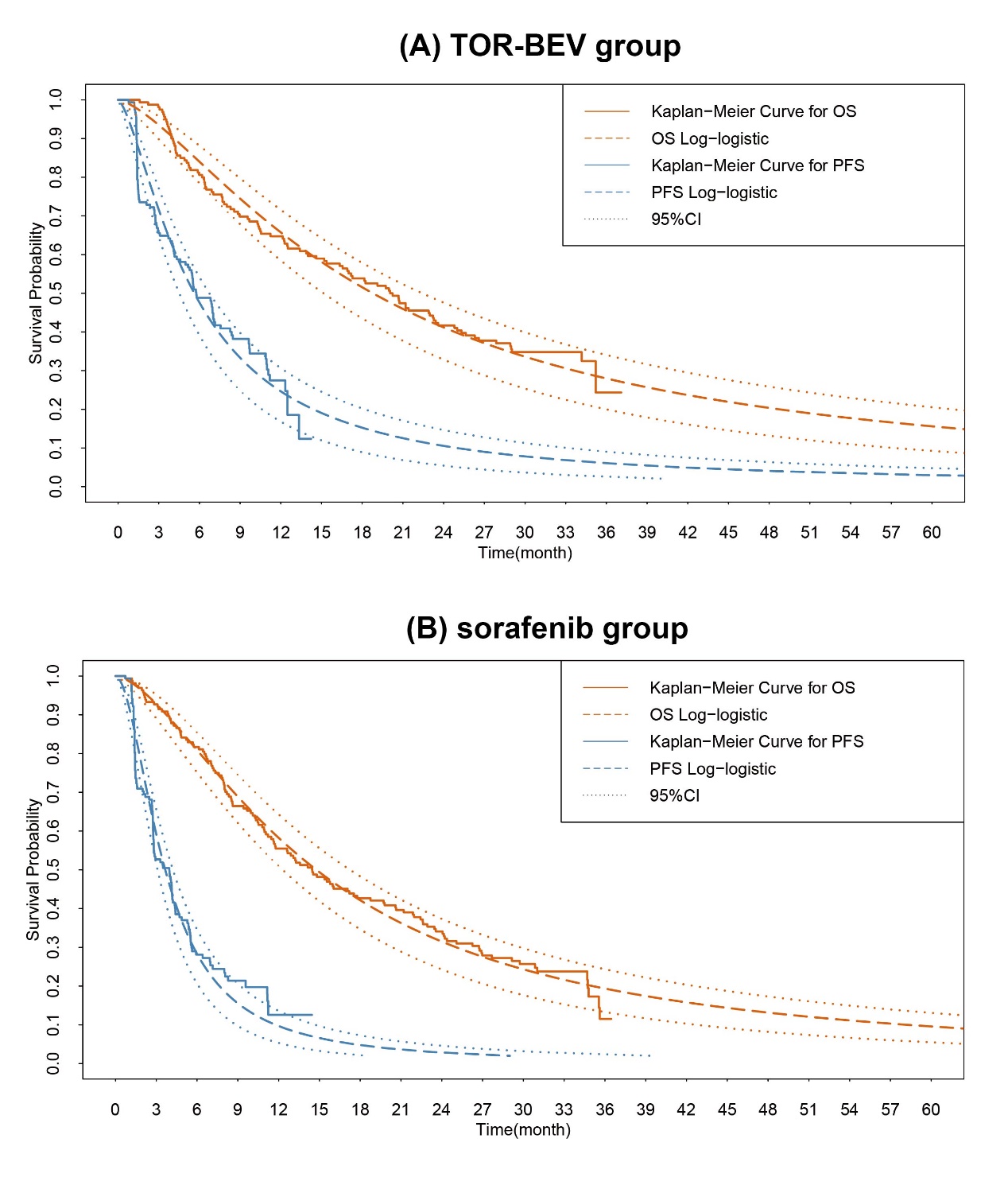


CI, confidence interval; OS, overall survival; PFS, progression-free survival; TOR-BEV, toripalimab plus bevacizumab.
